# Exploring the Mechanisms of Sanguinarine in the Treatment of Osteoporosis by Integrating Network Pharmacology Analysis and Deep Learning Technology

**DOI:** 10.2174/0115734099282231240214095025

**Published:** 2024-02-21

**Authors:** Yonghong Tang, Daoqing Zhou, Fengping Gan, Zhicheng Yao, Yuqing Zeng

**Affiliations:** 1 Department of Orthopedics, The Sixth People’s Hospital of Zhuji, Zhuji, Zhejiang, China;; 2 Department of Orthopedics, Pan’an Hospital of Traditional Chinese Medicine, Jinhua, Zhejiang, China;; 3 The First Clinical Medical College, Guangzhou University of Chinese Medicine, Guangzhou, Guangdong, China;; 4 Department of Orthopedics, Tongde Hospital of Zhejiang Province, Hangzhou, Zhejiang, China;; 5 The Second Clinical Medical College, Zhejiang Chinese Medical University, Hangzhou, Zhejiang, China

**Keywords:** Osteoporosis, network pharmacology analysis, sanguinarine, deep learning technology, protein-protein interaction network, molecular docking

## Abstract

**Background:**

Sanguinarine (SAN) has been reported to have antioxidant, anti-inflammatory, and antimicrobial activities with potential for the treatment of osteoporosis (OP).

**Objective:**

This work purposed to unravel the molecular mechanisms of SAN in the treatment of OP.

**Methods:**

OP-related genes and SAN-related targets were predicted from public databases. Differential expression analysis and VennDiagram were adopted to detect SAN-related targets against OP. Protein-protein interaction (PPI) network was served for core target identification. Molecular docking and DeepPurpose algorithm were further adopted to investigate the binding ability between core targets and SAN. Gene pathway scoring of these targets was calculated utilizing gene set variation analysis (GSVA). Finally, we explored the effect of SAN on the expressions of core targets in preosteoblastic MC3T3-E1 cells.

**Results:**

A total of 21 candidate targets of SAN against OP were acquired. Furthermore, six core targets were identified, among which CASP3, CTNNB1, and ERBB2 were remarkably differentially expressed in OP and healthy individuals. The binding energies of SAN with CASP3, CTNNB1, and ERBB2 were -6, -6.731, and -7.162 kcal/mol, respectively. Moreover, the GSVA scores of the Wnt/calcium signaling pathway were significantly lower in OP cases than in healthy individuals. In addition, the expression of CASP3 was positively associated with Wnt/calcium signaling pathway. CASP3 and ERBB2 were significantly lower expressed in SAN group than in DMSO group, whereas the expression of CTNNB1 was in contrast.

**Conclusion:**

CASP3, CTNNB1, and ERBB2 emerge as potential targets of SAN in OP prevention and treatment.

## 1 INTRODUCTION

Osteoporosis (OP) is a systemic skeletal condition marked by compromised bone tissue microstructure, decreased bone mass, increased fracture susceptibility, and heightened bone fragility [1-3]. It predominantly affects adults, especially postmenopausal women [4], with an insidious onset that can result in disability [5]. Many cases are asymptomatic in the early and middle stages, however, acute osteoporotic fractures will result in lifelong disability [6], resulting in increased mortality and a drastic reduction in the life quality of patients [7]. Furthermore, OP may trigger additional diseases, causing a significant financial burden on both the family of patients and society [8, 9]. Over the past few years, numerous studies have investigated the effectiveness of traditional Chinese medicine (TCM) for treating OP from a molecular biology perspective [10, 11]. Substantial advancements have been achieved [12, 13], providing additional evidence for the beneficial impact of TCM on OP treatment.

Sanguinarine (SAN, C_20_H_14_NO_4_^+^) is the most versatile benzophenanthridine alkaloid, extracted from the *Sanguinaria canadensis* and other poppy-fumaria species belonging to the Papaveraceae family [14]. SAN has been reported to possess not only antimicrobial [15], antioxidant [16], and anti-inflammatory [17] activities, but also have toxic and inhibitory effects on gastric cancer [18], lung cancer [19], and hepatocellular carcinoma [20] cells. Yu *et al.* identified SAN as a potential drug candidate for the treatment of OP through bioinformatics analyses [21]. Ma *et al.* revealed that SAN safeguarded mice against ovariectomy-induced OP through the modulation of bone remodeling, suggesting that SAN has potential application in the treatment of OP [22]. Zhang *et al*. found that SAN induced preosteoblastic MC3T3-E1 cell differentiation by activating the AMPK/Smad1 signaling pathway [23]. However, the molecular mechanism of SAN acting on OP still needs to be reported.

Network pharmacology, a burgeoning research methodology, combines the fundamental principles of systems biology with the latest developments in computer technology, and its inclusive and collaborative characteristics are in harmony with the principles of TCM [24, 25]. Therefore, by combining TCM with network pharmacology, a novel method, TCM network pharmacology emerged. This approach efficiently prioritizes disease-related genes, uncovers drug-gene-disease associations, and illustrates the network regulatory effects of TCM drugs [26]. Based on these facts, this work aimed to unravel the molecular mechanisms of SAN in treating OP using network pharmacology analysis. Furthermore, molecular docking and deep learning technologies were used to explore the interaction between SAN and the key targets. In addition, the qRT-PCR was utilized to investigate the effect of SAN on mouse preosteoblastic MC3T3-E1 cell lines.

## 2 MATERIALS AND METHODS

### 2.1 Screening Target Genes for SAN

The Herbal Ingredients' Targets Platform (HIT) [27], SwissTargetPrediction [28], SuperPred [29], Comparative Toxicogenomics Database (CTD) [30], and Genecards [31] online platforms were employed to predict the possible target genes for SAN.

### 2.2 OP-related Genes Acquisition

The Genecards, DisGeNET [32], and CTD online websites were employed to acquire OP-related genes. Notably, the genes obtained from the CTD database were sorted by Inference Score and only the top 20% were retained.

### 2.3 Differentially Expressed Genes (DEGs) and Candidate **Targets Selection**

Transcriptome expression profiling from GSE7158 was retrieved from the Gene Expression Omnibus (GEO, http://www.ncbi.nlm.nih.gov/geo/) platform [33] with 12 OP cases and 14 healthy individuals. R package limma [34] was adopted to filter out DEGs with *p* < 0.05 and |log fold change (FC)| > 0.2. VennDiagram [35] was further used for selecting candidate targets of SAN against OP by intersecting target genes for SAN, OP-related genes, and DEGs.

### 2.4 Gene Set Variation Analysis (GSVA)

A total of 19 signaling pathways related to the Wnt signaling pathway were detected from the MSigDB database [36]. Wnt signaling pathway scores of healthy individuals and OP cases from the GSE7158 dataset were assessed using the R package GSVA [37]. The t-test was further utilized to identify signaling pathways with significant differences in pathway scoring between OP and healthy samples. *p* < 0.05 was considered as the threshold.

### 2.5 Protein-protein Interaction (PPI) Network Establishment and Topological Analysis

Search Tool for the Retrieval of Interacting Genes (STRING) [38] online website was employed to predict the interactions of candidate targets and their interacting proteins. The filter is score_cutoff > 0.15 and size_cutoff < 20. The Cytoscape software [39] was utilized to build a PPI network. Subsequently, the core genes were filtered by taking the intersection of the top 10 genes screened using Betweenness Centrality (BC), Closeness Centrality (CC), Degree, and Radiality algorithms in Cytoscape. GSE35958 dataset with 5 OP cases and 4 healthy individuals was utilized to verify the expression of core genes. Core genes that were significantly differentially expressed in OP cases and healthy individuals were utilized for followed analysis.

### 2.6 Drug-target Binding Capacity Prediction

We obtained 3D protein structures of core targets from the RCSB Protein Data Bank (RCSB PDB, http://www.pdb.org/) [40]. The obtained structures were then processed using Pymol software to isolate modified regions, ligands, and remove water molecules. Next, the 3D structure of SAN was acquired from PubChem (https://pubchem.ncbi.nlm.nih.gov/) [41], and charges were calculated for the molecule after adding hydrogen atoms. The docking process was performed using AutoDock 4.2.6 software [42] and identified potential binding sites, and limited binding energy < -5.0 kcal/mol indicating stable binding sites [43].

DeepPurpose (https://github.com/kexinhuang12345/DeepPurpose) serves as a deep learning library specifically crafted for predicting drug-target interactions (DTI), which facilitates swift model building [44]. Therefore, DeepPurpose was employed to further validate the binding ability of core targets and the SAN. For SAN, the encoder employed was Multi-Layer Perceptrons (MLP) on Morgan; for the amino acid sequences of the target proteins, the encoder used was MLP on Amino Acid Composition (AAC). Ultimately, the binding scores of SAN with the key targets were computed using the Morgan_AAC_DAVIS model. The simplified molecular-input line-entry system (SMILES) of SAN was obtained from PubChem, and all amino acid sequences of the key targets were retrieved from UniProt (https://www.uniprot.org/) [45].

### 2.7 Cell Culture

Mouse preosteoblastic MC3T3-E1 cell lines were purchased from Yaji Biological Co. (Shanghai, China). Subsequently, the cell lines were cultured in α-minimal essential medium (α-MEM, Gibco, NY, USA) medium containing 10% fetal bovine serum (FBS, Gibco, USA), 100 μg/mL streptomycin, and 100 U/mL penicillin, and then incubated at 37°C and 5% CO_2_ for 2-3 days in a humid environment. In addition, it was routinely passaged when cell density was approximately 80-90%. Cells were inoculated in 6-well plates at a density of 2×10^5^ cells per well for 24 h. After the cells were attached to the well, they were treated with SAN (2 μM, Yuanye Bio-Technology Co., Ltd, Shanghai, China) or vehicle (DMSO, Sigma-Aldrich, MO, USA) for 48 h. An alkaline phosphatase (ALP) colorimetric assay kit (Elabscience, Wuhan, China) was applied to determine the ALP activity at 48 h after treatment according to the manufacturer’s instructions.

### 2.8 Quantitative Reverse Transcription Polymerase Chain Reaction (qRT-PCR)

Total RNA was extracted using Trizol Reagent (Invitrogen, CA, USA). Extracted RNA was converted to cDNA using the RevertAid First Strand cDNA synthesis kit (TIANGEN, German) according to the manufacturer’s instructions. The qRT-PCR was conducted using CFX96 Touch Real-Time PCR Detection System (Bio-Rad, CA, USA). The expression levels of core genes were quantified employing the 2^-ΔΔCT^ methodology, and GAPDH was utilized as an internal control. Primers are exhibited in Table **1**.

### 2.9 Statistical Analysis

Bioinformatics analyses were performed using R (Version 4.2) and python softwares. Experimental data analyses were done using GraphPad 7.0 software and *p* < 0.05 was the cutoff value. All experimental data are presented as mean ± standard deviation (Mean ± SD), and differences between groups were compared by One-way ANOVA, followed by Tukey’s post hoc test.

## 3 RESULTS

### 3.1 Acquisition of Candidate Targets of SAN against OP and GSVA Analysis

**Fig. (1):**
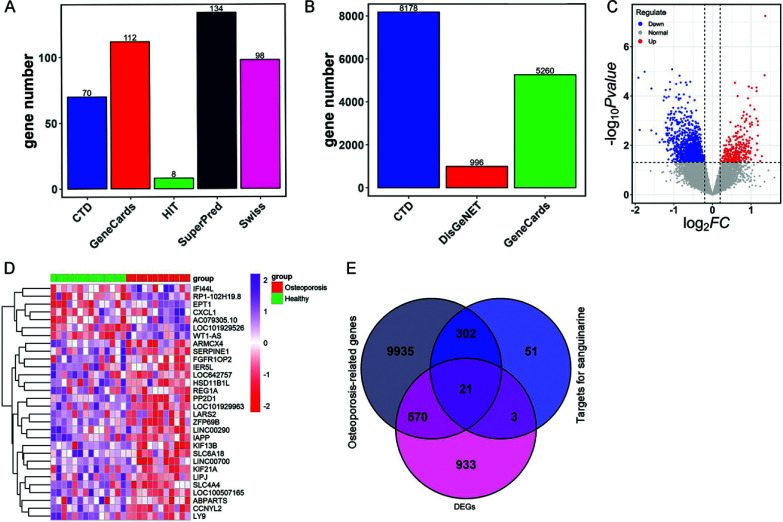
Identification of targets of SAN against OP. (**A**) SAN-related targets obtained from different databases. (**B**) OP-related genes obtained from different databases. (**C**) Volcano map of DEGs between healthy and OP samples. Blue: down-regulation; red: up-regulation. (**D**) A heatmap of the expression of top 30 DEGs in each sample. (**E**) Venn diagram showed 21 intersecting genes among SAN-related targets, OP-related genes, and DEGs. **Abbreviations:** SAN: sanguinarine; OP: osteoporosis; DEGs: differentially expressed genes; HIT: Herbal Ingredients' Targets Platform; CTD: Comparative Toxicogenomics Database.

After deleting duplicates, 377 targets for SAN and 10,828 OP-related genes were obtained from public databases (Figs. **1A** and **B**, Table **S1**). A total of 1,527 DEGs between healthy individuals and OP cases in the GSE7158 dataset were conformed, consisting of 1247 down-regulated genes and 280 up-regulated genes (Fig. **1C**, Table **S2**). The heatmap exhibited the expressions of the top 30 DEGs (Fig. **1D**). Subsequently, 21 candidate targets of SAN against OP were acquired by overlapping 377 targets for SAN, 10,828 OP-related genes, and 1,527 DEGs (Fig. **1E**, Table **S3**).

### 3.2 PPI Network Development and Core Targets Identification

**Fig. (2):**
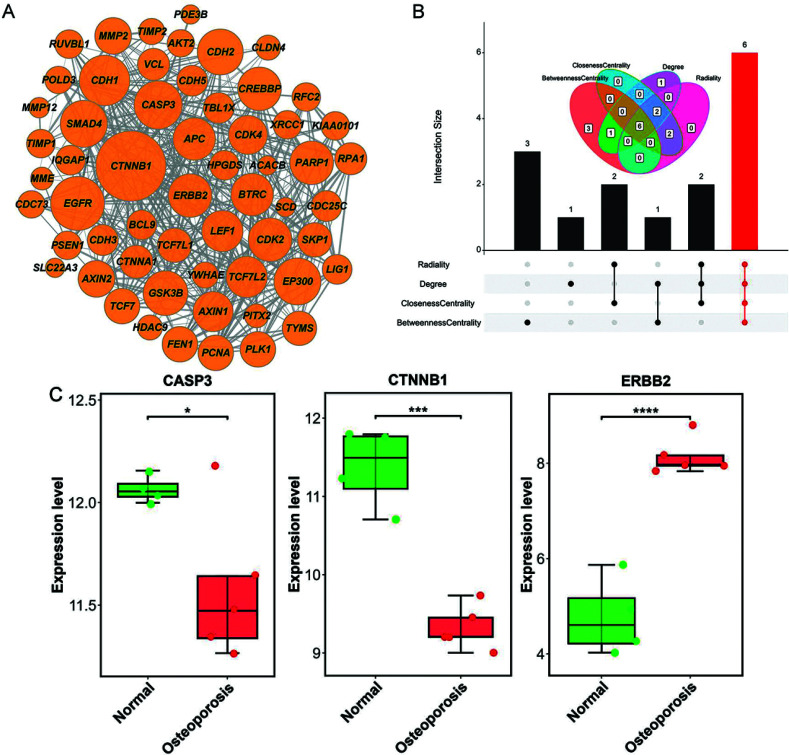
Identification of core targets of SAN against OP. (**A**) PPI network based on 21 candidate targets. (**B**) Topological analysis based on PPI network. (**C**) The expression of CASP3, CTNNB1, and ERBB2 between healthy and OP sample in GSE35958. *p < 0.05; ***p < 0.001; ****p < 0.0001; **Abbreviations:** SAN: sanguinarine; OP: osteoporosis; PPI: protein-protein interaction.

The identified 21 candidate targets were entered into STRING to eliminate unconnected targets, and a PPI network with 404 pairs of interactions was further developed (Fig. **2A**). Subsequently, six core targets (CTNNB1, EGFR, EP300, CASP3, CDH1, and ERBB2) were identified by intersecting the top 10 genes obtained from the network topology parameters BC, CC, Degree and Radiality (Fig. **2B**). Furthermore, we found that the CASP3 (*p* < 0.05) and CTNNB1 (*p* < 0.001) were remarkably lower expressed in OP samples than in healthy individuals, whereas ERBB2 were remarkably higher expressed in OP cases than in healthy individuals in the GSE35958 dataset (*p* < 0.0001, Fig. **2C**).

### 3.3 Molecular Docking Analysis and GSVA

**Fig. (3):**
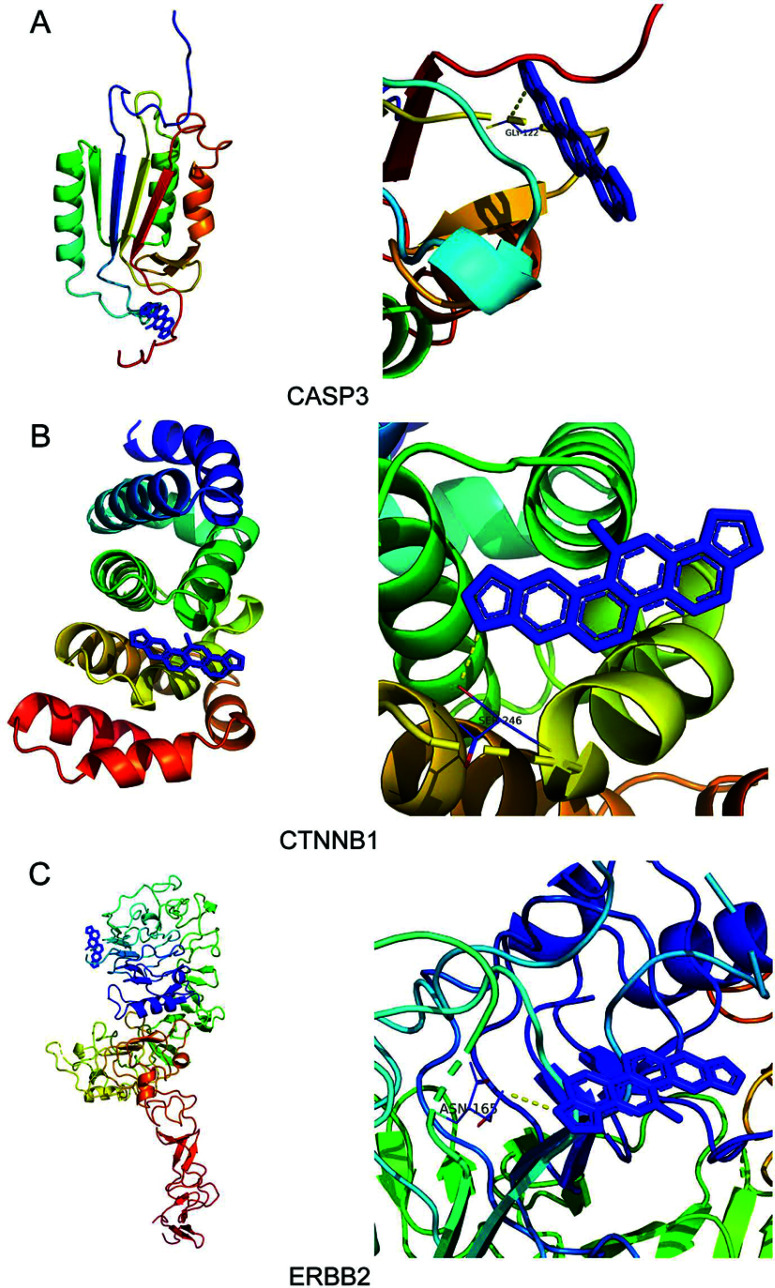
Molecular docking site. (**A**) CASP3-SAN. (**B**) CTNNB1-SAN. (**C**) ERBB2-SAN. **Abbreviation:** SAN: sanguinarine.

**Fig. (4):**
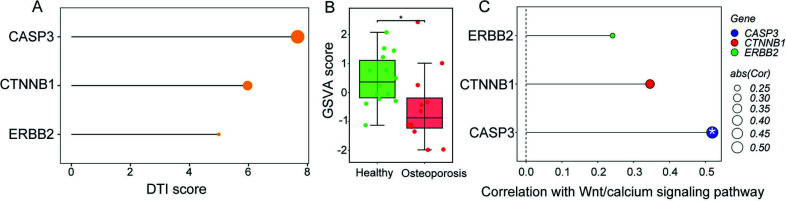
Validation of the binding ability of core targets and SAN and GSVA analysis. (**A**) CASP3 and SAN had the highest binding score. (**B**) Box plot showed the GSVA score of the Wnt/calcium signaling pathway between OP cases and healthy individuals. (**C**) Pearson correlation analysis of core targets and Wnt/calcium signaling pathway. Cor: coefficient. *p < 0.05; **Abbreviations:** GSVA: gene set variation analysis; SAN: sanguinarine; OP: osteoporosis.

Molecular docking was employed to explore the binding of SAN and three core targets (CASP3, CTNNB1, and ERBB2). Typically, the binding energy below -5.0 kcal/mol indicates favorable binding affinity, and the binding energy below -7.0 kcal/mol signifies strong binding affinity [46]. The binding energies of SAN with CASP3, CTNNB1, and ERBB2 were -6, -6.731, and -7.162 kcal/mol, respectively (Figs. **3A**-**C**, Table **2**). The consequences suggested that the identified core targets indeed bind stably to SAN. In addition, DeepPurpose was further used to predict and validate the binding ability of core targets and SAN. The result demonstrated that CASP3 and SAN had the highest binding scores (Fig. **4A**). Moreover, the Wnt signaling pathway closely associates various cell types, including bone marrow mesenchymal stem cells (BMSCs), osteoclasts, osteoblasts, and chondrocytes [47]. Therefore, we conducted GSVA analysis, and the result demonstrated that GSVA scores of the Wnt/calcium signaling pathway were significantly lower in OP cases compared to healthy individuals (*p* = 0.036, Fig. **4B**, Table **3**). Moreover, the Pearson correlation method was adopted to explore the relationships between the expression levels of these three core targets and Wnt/calcium signaling pathway. We found that the expression level of CASP3 was positively associated with Wnt/calcium signaling pathway (*p* < 0.05, correlation coefficient > 0.5) (Fig. **4C**).

### 3.4 The Effects of SAN on the Expression of Core Targets by qRT-PCR

**Fig. (5):**
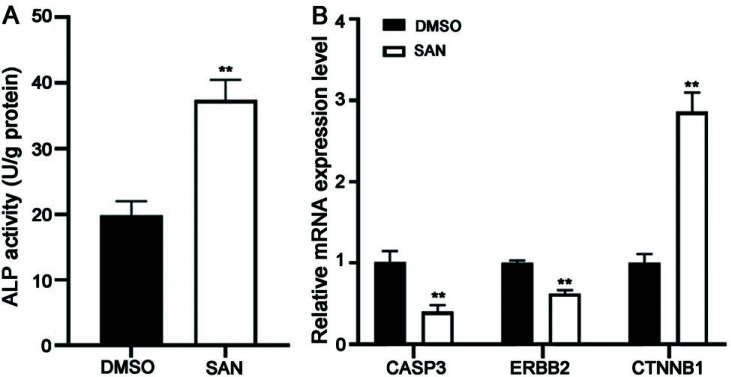
Investigation of the effects of SAN on the expression of core targets in preosteoblastic MC3T3-E1 cells by qRT-PCR. (**A**) ALP activity between DMSO and SAN groups in the preosteoblastic MC3T3-E1 Cells. (**B**) The Expression Levels of CASP3, ERBB2, and CTNNB1 between DMSO and SAN groups in the preosteoblastic MC3T3-E1 cells. MC3T3-E1 cells were treated with 2 μM SAN or DMSO. **p < 0.01; **Abbreviations:** DMSO: vehicle; SAN: sanguinarine; qRT-PCR: quantitative reverse transcription polymerase chain reaction; ALP: alkaline phosphatase.

To investigate the effects of SAN on the expression of core targets, qRT-PCR based on mouse preosteoblastic MC3T3-E1 cell lines was conducted. ALP serves as a clear marker for osteoblastic differentiation [48], therefore, we determined the ALP activity between SAN and DMSO groups. The results demonstrated that ALP activity levels were elevated in the group treated with SAN compared to the group treated with DMSO (*p* < 0.01, Fig. **5A**). The expression of CASP3 (*p* < 0.01) and ERBB2 (*p* < 0.01) were remarkably lower in the SAN group than in the DMSO group (Fig. **5A**). CTNNB1 was remarkably higher expressed in the SAN group than in the DMSO group (*p* < 0.01, Fig. **5B**).

## 4 DISCUSSION

SAN has been reported to inhibit osteoclast formation and bone resorption by inhibiting extracellular signal-regulated kinase and nuclear factor-κB signaling pathways [49]. However, the molecular mechanisms of SAN against OP needs to be further explored. In the current work, we found that CASP3, CTNNB1, and ERBB2 were the core targets of SAN against OP. Besides, the three target genes have excellent binding properties (bind energy ≤ -5.0 kcal/mol) with SAN. Furthermore, we found ERBB2 was more expressed in OP cases than in healthy individuals in GSE35958 dataset, whereas the expression of levels of CASP3 and CTNNB1 were in contrast. Consistently, in mouse preosteoblastic MC3T3-E1 cells, the expression levels of CASP3 and ERBB2 were lower whereas the expression of CTNNB1 was higher in SAN group than in DMSO group. The results indicated that SAN could suppress the expression of CASP3 and ERBB2, and promote the expression of CTNNB1.

ERBB2, also known as HER2, played a crucial role in cell differentiation and proliferation [50]. In addition, ERBB2 signaling was involved in developing, growing, and repairing bones [51]. Frequent disruption of Wnt signaling often arises from aberrant CTNNB1 expression, the gene responsible for encoding β-catenin [52]. Accumulating evidence shows that abnormal CTNNB1 is implicated in pathogenesis [53-55]. CTNNB1 has been recognized as a participant in influencing both bone mineral density (BMD) and the predisposition to OP [56]. Furthermore, CTNNB1 can regulate osteoclastogenesis and osteoblastic differentiation [57]. Hasan *et al.* found CTNNB1 and ERBB2 were important genes involved in the pathogeneses of OP [58]. ERBB2 played a crucial role in the pathogenesis of postmenopausal OP, promoting bone resorption and negatively affecting bone formation [58]. ERBB2 affects bone remodeling processes, including the formation and activity of bone remodeling units, by regulating the Wnt/β-catenin signaling pathway [59]. CASP3 plays a vital role in infiltration, programmed cell death, and inflammatory diseases [60, 61]. CASP3 plays a vital role in the initiation and termination phases of apoptosis, and its activation shows the irreversible phase of apoptosis [62, 63]. Moreover, CASP3 serves as a crucial anti-tumor target since its cleavage and activation trigger apoptosis, ultimately resulting in tumor cell death, making its activators promising anticancer drugs [64]. Prior studies revealed that SAN could trigger tumor cell apoptosis through CASP3 activation to exert anticancer effects [65-68]. However, a recent study has indicated that decreased CASP3 expression induced by miR-758-3p can suppress osteoblast apoptosis, thereby alleviating OP [69]. These studies suggest the complex roles of CASP3 in the therapeutic mechanisms of sanguinarine against different diseases. Therefore, the specific anti-OP mechanisms of SAN aby regulating CASP3 needs to be further elucidated in subsequent studiesWe found ERBB2, CTNNB1, and CASP3 have the potential to be therapeutic targets for SAN against OP, and qRT-PCR results suggested that CASP3 and ERBB2 were significantly lower expressed in the SAN group than in the DMSO group, whereas the opposite was true for CTNNB1. Therefore, SAN may inhibit the progression of OP by suppressing the expression of CASP3 and ERBB2. Besides, SAN may affect osteoclastogenesis and osteoblast differentiation by targeting CTNNB1, thereby treating OP.

The Wnt signaling pathway is involved not only in bone development and metabolism, but also in the proliferation and differentiation of chondrocytes, mesenchymal stem cells, osteoblasts, and osteoclasts [70]. Current anabolic treatments for OP target the Wnt signaling pathway [71]. The Wnt signaling pathway has been found to be correlated to the formation and development of OP [72-74]. The Wnt gene is correlated to the differentiation, growth, and apoptosis of mesenchymal stem cells, and is vital for the regulation of bone development, formation, and signaling in OP [75]. We found that the GSVA score of the Wnt/calcium signaling pathway was significantly lower in OP cases than in healthy individuals. In the Wnt/calcium signaling pathway, Wnt5a binding to the frizzled receptor activates NFAT and NFκB, and influences osteoclastogenesis [76]. Pearson correlation analysis revealed a significant positive correlation between CASP3 and Wnt/calcium signaling pathway activity scores (*p* < 0.05). Upon the interaction between a Wnt protein and a cell surface receptor, a cascade of molecular signals is triggered within the target cell. This interaction with activated receptors subsequently induces an elevation in intracellular calcium levels and prompts the activation of protein kinase C [77]. Kuncewitc *et al.* found that the Wnt agonist reduced cleaved CASP3 by 46%, suggesting remission of hemorrhage-induced apoptosis in the lungs [78]. However, the mechanism of Wnt/calcium signaling pathway in OP has not yet been reported, and it needs to be further investigated.

This study has some limitations. Firstly, our findings largely depended on public databases that need to be updated real time, which may neglect some other crucial pathways or targets. Secondly, the affinities of SAN with the core targets were evaluated using the existing model that has its own limitations. Thirdly, we merely verified the therapeutic effects and its regulatory roles in the key targets at the *in vitro* level without *in vivo* validation. Therefore, future research should include broader experiments to further expound the core mechanisms underlying SAN treating OP. Besides, more clinical trials are needed to validate the role of SAN in OP.

## CONCLUSION

In this investigation, we employed network pharmacology, bioinformatics analysis, and molecular docking to reveal the mechanisms of SAN against OP. CASP3, CTNNB1, and ERBB2 emerge as potential targets through which SAN may exert preventive and therapeutic effects on OP. These findings provide novel insights into the molecular mechanisms underlying the beneficial impacts of SAN in addressing OP, suggesting SAN has potential as a treatment for OP.

## Figures and Tables

**Table 1:** Primer sequences for qRT-PCR.

**Primer**	**Forward Primer Sequence (5’-3’)**	**Reverse Primer Sequence (5’-3’)**	**Size (bp)**
ERBB2	TCCCCAGGGAGTATGTGAGG	GAGGCGGGACACATATGGAG	707
CASP3	CTCGCTCTGGTACGGATGTG	TCCCATAAATGACCCCTTCATCA	201
CTNNB1	ATGGAGCCGGACAGAAAAGC	TGGGAGGTGTCAACATCTTCTT	143
GAPDH	TGGCCTTCCGTGTTCCTAC	GAGTTGCTGTTGAAGTCGCA	178

**Table 2:** Molecular docking binding energy.

**Target**	**Uniprot ID**	**PDB 1ID**	**PubChem ID**	**Compound**	**Free Binding Energy (kcal/mol)**
CASP3	P42574	2xyg	5154	Sanguinarine	-6
CTNNB1	P35222	7afw			-6.731
ERBB2	P04626	1n8z			-7.162

**Table 3:** The t-test was performed on the GSVA scores of 17 Wnt-related signaling pathways between healthy and osteoporosis samples from the GSE7158 dataset.

**Pathway**	**Group 1**	**Group 2**	***p***	**Method**
Willert Wnt signaling	Healthy	Osteoporosis	0.408	t-test
Wnt/calcium signaling pathway			**0.036**	
Regulation of Wnt signaling pathways			0.287	
Non-canonical Wnt signaling pathway			0.245	
Non-canonical Wnt signaling pathway *via* MAPK cascade			0.082	
Canonical Wnt signaling pathway			0.266	
Regulation of canonical Wnt signaling pathway			0.260	
Wnt signalosome			0.655	
Wnt protein binding			0.863	
Wnt receptor activity			0.965	
Hallmark Wnt beta catenin signaling			0.279	
Reactome signaling by Wnt			0.409	
WP Wnt signaling			0.358	
Biocarta Wnt pathway			0.311	
KEGG Wnt signaling pathway			0.351	
PID Wnt signaling pathway			0.444	
Wnt signaling			0.296	

**Note:** Bold indicates *p* < 0.05.

## Data Availability

The data that support the findings of this study are available from the corresponding author, [YZ], on special request.

## LIST OF ABBREVIATIONS

AAC: Amino Acid Composition
ALP: Alkaline Phosphatase
BC: Betweenness Centrality
BMD: Bone Mineral Density
CC: Closeness Centrality
CTD: Comparative Toxicogenomics Database
DEGs: Differentially Expressed Genes
DTI: Drug-target Interaction
FC: Fold Change
GEO: Gene Expression Omnibus
GSVA: Gene Set Variation Analysis
HIT: Herbal Ingredients' Targets Platform
MLP: Multi-Layer Perceptrons
OP: Osteoporosis
PPI: Protein-protein Interaction
qRT-PCR: Quantitative Reverse Transcription Polymerase Chain Reaction
RCSB PDB: RCSB Protein Data Bank
SAN: Sanguinarine
SMILES: Simplified Molecular-input line-entry System
STRING: Search Tool for the Retrieval of Interacting Genes
TCM: Traditional Chinese Medicine

## SUPPLEMENTARY MATERIAL

Supplementary material is available on the publisher’s website along with the published article. Supplementary Table 1. Targets for sanguinarine and osteoporosis-related genes were obtained from online databases. Supplementary Table 2. Differentially expressed genes between healthy individuals and osteoporosis cases in GSE7158 dataset. Supplementary Table 3. A total of 21 intersecting targets.
