# Supplementary material for: Exploring the Mechanisms of Sanguinarine in the Treatment of Osteoporosis by Integrating Network Pharmacology Analysis and Deep Learning Technology
Source: Curr Comput Aided Drug Des. 2024 Feb 21;21(1):83–93. doi: 10.2174/0115734099282231240214095025 (PMC11774308; doi:10.2174/0115734099282231240214095025)
Supplement: Supplementary file 1 — Supplementary material is available on the publisher’s website along with the published article. Supplementary Table 1. Targets for sanguinarine and osteoporosis-related genes were obtained from online databases. Supplementary Table 2. Differentially expressed genes between healthy individuals and osteoporosis cases in GSE7158 dataset. Supplementary Table 3. A total of 21 intersecting targets. [file CCADD-21-1-83_SD1.zip › CCADD-SUPPLY-2023-233.pdf]

SUPPLEMENTARY MATERIAL

Exploring the Mechanisms of Sanguinarine in the Treatment of Osteoporosis by Integrating Network Pharmacology Analysis and Deep Learning Technology

Yonghong Tang[1], Daoqing Zhou[2], Fengping Gan[3], Zhicheng Yao[4] and Yuqing Zeng[3,4,5,*]

[1]Department of Orthopedics, The Sixth People's Hospital of Zhuji, Zhuji, Zhejiang, China; [2]Department of Orthopedics, Pan'an Hospital of Traditional Chinese Medicine, Jinhua, Zhejiang, China; [3]The First Clinical Medical College, Guangzhou University of Chinese Medicine, Guangzhou, Guangdong, China; [4]Department of Orthopedics, Tongde Hospital of Zhejiang Province, Hangzhou, Zhejiang, China; [5]The Second Clinical Medical College, Zhejiang Chinese Medical University, Hangzhou, Zhejiang, China
